# The Mediating Role of the Patient Health Engagement Model on the Relationship Between Patient Perceived Autonomy Supportive Healthcare Climate and Health Literacy Skills

**DOI:** 10.3390/ijerph17051741

**Published:** 2020-03-07

**Authors:** Serena Barello, Lorenzo Palamenghi, Guendalina Graffigna

**Affiliations:** EngageMinds Hub – Consumer, Food & Health Engagement Research Center, Department of Psychology, Università Cattolica del Sacro Cuore, L.go Agostino Gemelli 1, 20143 Milano, Italy; lorenzo.palamenghi@unicatt.it (L.P.); guendalina.graffigna@unicatt.it (G.G.)

**Keywords:** health literacy, patient health engagement model, Health Care Climate Questionnaire, patient autonomy, PHE-S, Patient Health Engagement Scale, health communication, patient-centered communication, patient engagement

## Abstract

Individuals with low health literacy (HL) are known to have poorer health outcomes and to have higher mortality rates compared to individuals with higher HL; hence, the improvement of HL is a key outcome in modern healthcare systems. Healthcare providers are therefore asked to support patients in becoming more and more engaged in their healthcare, thus augmenting their literacy skills. Our main hypothesis is that the well-known relationship between patients’ perceived autonomy supportive healthcare climate and HL skills is mediated by the Patient Health Engagement Model (PHE-model) which describes the patients’ progressive maturation of a psychological readiness to become active players in their healthcare. The purpose of this study was to formulate a hypothetical structural equation model (SEM) linking an autonomy-supportive healthcare climate to PHE-model and HL. A cross-sectional survey design was employed involving 1007 Italian chronic patients. The hypothetical model was tested using SEM to verify the hypothesized mediation of the PHE-model between autonomy-supportive healthcare climate and HL. Results show that the theoretical model has a good fit indexes and that PHE-model fully mediates the relationship between autonomy-supportive healthcare climate and HL. This finding suggests healthcare systems to implement a new paradigm where patients are supported to play an autonomous role in their own healthcare.

## 1. Introduction

Health literacy is becoming a focal issue for health providers and policy makers in many countries around the world. Health literacy is defined as “the degree to which individuals have the capacity to obtain, process, and understand basic health information and services needed to make appropriate health decisions” [1,2,3]. According to research, individuals with lower health literacy are more likely to have poor health outcomes, are less likely to understand their health problems and care management, and are at higher risk of hospitalizations and mortality rates [4,5]. Finally, the healthcare costs associated with low heath literacy are estimated at $50 to $73 billion annually [6]. Therefore, improving patients’ level of health literacy is a major goal of the World Health Organization. According to these premises, the identification of the factors that may affect patients’ health literacy is crucial to answer to the question “How do patients become health literate regarding their health condition?” and to develop interventions aimed at sustaining this skill. 

## 2. Theory and Hypotheses

To address the burden of patients’ limited health literacy, healthcare systems should redesign their services to support patients to effectively navigate, understand, and use information to take care of their health [7,8,9]. This transformation can be accomplished by encouraging healthcare providers to embrace patient engagement as a guiding paradigm to foster the patient’s proactivity and autonomy in obtaining and using health information for an effective management of their care. However, to become fully engaged in their healthcare, patients should acquire a “psychological readiness” towards the possibility to take an active and autonomous role in their own healthcare. This psychological maturation is function of the patients’ emotional elaboration of their health condition and of their new role identity (i.e., as a patient). This process has been widely described by the Patient Health Engagement Model (PHE-model) [10] that points to the role of the patients’ emotional maturation as the main driver of their ability to adjust to the changes required by the new health condition. The psychological process of engagement as described by the PHE-model involves four developmental phases, namely, blackout, arousal, adhesion, and eudaimonic project. The PHE-model theorizes possible psychological trajectories of engagement along a continuum from a position of disengagement (namely, blackout), where patients experience feelings of psychological vulnerability connected to the illness and feel psychologically frozen and feel paralyzed, to a position of full engagement (namely, eudaimonic project) where patients become totally aware of their disease and its implications and have elaborated and accepted their “new identity” of patient and became able to embrace a more positive and satisfactory approach to their life [11,12,13,14]. In this scenario, to foster the patient’s emotional elaboration, of much relevance is the ability of healthcare professionals to promote a healthcare climate which is supportive of the patient’s autonomy, which has been associated with numerous positive patient outcomes [15]. Research demonstrated that an autonomy-supportive healthcare climate of mutual understanding, trust, and shared decision-making has been found to improve healthcare outcomes by increasing PHE [16,17]. In healthcare encounters, the concept of autonomy support represents a relational climate whereby the healthcare professionals put patients at the center of their care experience, enabling them to become fully engaged along the care journey [18,19].

Therefore, the implementation of such model of care oriented to a participatory style in the relationship with patients forces professionals to move back from a disease-centered approach toward a patient-engagement-oriented one which encompasses the key principles of patient-centered care by also implying the consideration of patients as partners. This means building good interpersonal relationships among patients and their health providers by increasing trust, facilitating information exchange, and supporting patients’ autonomy by recognizing and cultivating their own capabilities and self-management skills as individuals who have to reconstruct a “new normality” around the disease condition [20,21].

According to self-determination theory, patients’ sense of autonomy represents a critical component of their motivational profile for effective self-care [18,22,23]. In healthcare settings, autonomous motivation is of particular importance to improve health literacy levels [24,25,26], and an autonomy-supportive healthcare climate is, therefore, a crucial strategy for achieving such goal [27].

Based on the literature above, our first hypothesis posited that:

**Hypothesis** **1.** *There is a significant, positive relationship between patient perceptions of autonomy-supportive healthcare climate and health literacy levels*.

According to the literature described above, the Patient Health Engagement model refers to the patient’s psychological readiness to play an active role in his/her own healthcare journey as an autonomous actor taking increased responsibility for decision-making regarding his or her health. [28]. This model is rooted in the concept of patients’ autonomy, and thus it values the importance of supporting patients’ interest in and desire to participate in healthcare decisions. In this vein, PHE is a motivational and psychological-rooted model and does not involve the patient as a passive recipient of information, whose task is the mere comprehension and acceptance of information. In these terms, PHE is a crucial factor for improving patients’ health literacy in terms of collecting, understanding, and using information about their health condition [1,29,30]. Research demonstrated that patients improve their health literacy to a point where they become more involved in healthcare processes (including shared decision-making) [31]. If patients are not psychologically ready to assume an active role in their healthcare and are not supported in playing an autonomous role in their healthcare, they cannot obtain, process, and understand basic health information that is useful to take care of themselves well or make good decisions on health [32].

Based on the literature above, we next posit that:

**Hypothesis** **2.** *There will be a significant, positive relationship between PHE-model levels and health literacy*.

Moreover, previous research has demonstrated how the patient perception of autonomy support from their healthcare providers is related to increasing in the PHE positions [33,34,35]. A healthcare climate oriented to promote the patient ability to take an active and autonomous role in the care management has been pointed out as a predictor of the PHE positions [36,37,38]. The more that patients feel legitimized to be autonomous actors in their care management, the more they increase their psychological readiness to be engaged in their own care.

Based on the literature above, the third hypothesis posited that:

**Hypothesis** **3.** *There will be a significant, positive relationship between patient perceptions of patient reported autonomy supportive healthcare climate and PHE positions*.

**Figure 1:**
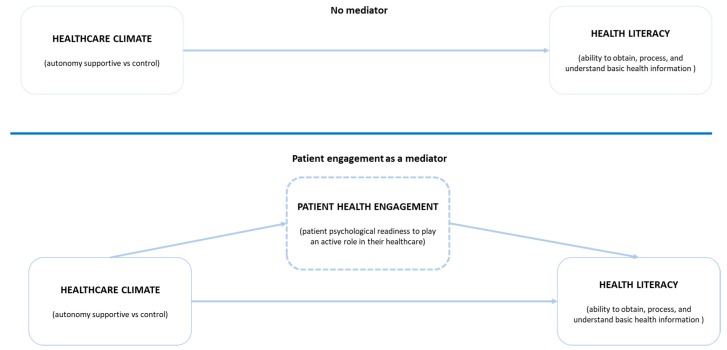
Model: the mediation effect of patient health engagement on healthcare climate in health literacy.

Although research and clinical practice suggests that an autonomy-supportive healthcare climate may be important to enhance the patients’ psychological readiness to engage and health literacy, no prior study, to the best of our knowledge, has developed and tested comprehensive models for capturing the relationship between these variables that have been included in our proposed theoretical model (see Figure 1).

For these reasons we added a fourth hypothesis that posited that:

**Hypothesis** **4.** *Psychological readiness to engage mediates the relationship between patient reported autonomy supportive healthcare climate which is supportive of patient’s autonomy and health literacy*.

To address this literature gap, our objectives were to: (1) examine the association between autonomy-supportive healthcare climate and health literacy levels; (2) examine the association between autonomy-supportive healthcare climate and patients’ readiness to engage measured with the PHE-model; (3) examine the association between PHE-model and health literacy; and (4) use mediation analysis to explore whether patient reported autonomy supportive healthcare climate might contribute to patients’ health literacy by supporting the psychological readiness to engage (PHE-model). 

## 3. Methodology

### 3.1. Study Design and Participants

To answer the research questions, this study applied a multistage, stratified sampling method to obtain a national-representative sample of adult patients with chronic disease. Eligibility criteria for being involved in the study were purposefully kept minimal to make the results broadly applicable and included having a chronic physical condition, being 18 years old and older, and being able to read and understand Italian. Patients were recruited from an online panel of patients with chronic conditions provided by Toluna (http://www.toluna.com). People belonging to the online panel were carefully screened for authenticity and legitimacy via digital fingerprint and geo-IP-validation from the panel provider. All panelists were profiled on the basis of their sociodemographic, clinical, and lifestyle characteristics. The panel was certified to be statistically representative of all the covered populations. In our study, in order to guarantee data quality, respondents were asked to confirm their demographics and health condition.

Qualtrics web-based survey service [39] was used to design the questionnaire, manage the survey, and collect data. A total of 2616 chronic patients accessed the questionnaire. Of these 2616 entries, 1609 were discarded because of uncomplete answers, resulting in 1007 complete entries which were kept for further analysis. The questionnaire took only a few minutes to be completed. 

### 3.2. Measures

To invoke the constructs inserted in our theoretical model, validated self-report scales were used in this study.

Patient reported autonomy supportive healthcare climate was assessed by the Health Care Climate Questionnaire (HCCQ) developed by Williams and colleagues [40]. This is a questionnaire aimed to capture the patient perception of the degree of centeredness featuring the patient–doctor communication defined as taking the patient’s perspective, encouraging and answering their questions, supporting their initiatives, offering choice about treatment options, and minimizing control. In this study we adopted the six-item version [41] that includes items 1, 2, 4, 7, 10 and 14 from the original 15-item version. Sample items include ‘I feel that my doctor has provided me choices and options’, ‘My doctor tries to understand how I see things before suggesting a new way to do things’, and ‘My doctor encourages me to ask questions’. Each item was rated on a 7-point Likert scale ranging from 1 (strongly disagree) to 7 (strongly agree). The language of the scale is devoid of jargon, double negative statements, and advanced vocabulary to optimize accessibility for individuals across education levels. Similarly to the original 15-item scale, the short version used in the current study showed a high internal consistency (Cronbach’s α = 0.96) and a 1-factor structure in the validation sample. 

Health literacy was measured through the Brief Health Literacy Screener (BHLS), a brief scale composed of three items that, according to the findings from Chew and colleagues [42], are effective in detecting the patients’ health literacy level. The BHLS has largely been validated in research and outpatient settings as a verbally administered three-item tool. Research has previously examined its utility in the inpatient setting compared to the REALM-R and demonstrated that the two tools did not find a similar prevalence of low HL among our inpatient study population [43] The three BHLS items assess literacy, interaction, comprehension, and confidence (self-efficacy) skills [44]. Answers are given using a 5-point Likert scale (0 = Never; 4 = Always).

Finally, the Patient Health Engagement Scale (PHE-S) [45] was used to assess the patient psychological readiness to take an active role in their healthcare. This scale was developed according to the Patient Health Engagement Model [10] which features four “positions” along a continuum of patient engagement (i.e., blackout, arousal, adhesion, eudaimonic project). PHE-S was specifically designed to assess the level of patients’ engagement, and it consists of five items surveying the patient’s experience of engagement in the care pathway. Answers are collected on a 7-point scale (lower scores meaning a patient engagement level closer to the “blackout” position, higher scores indicating a patient engagement level closer to “eudaimonic project”). The peculiarity of this scale is that it allows not only to assess the patient’s attitude towards his/her health condition, but also to forecast the patient’s risk for disengagement in disease management. Scoring is available upon request to the authors. 

### 3.3. Data Analysis

Descriptive statistics were performed using JASP v0.11.1 [46]. Means, standard deviations, skewness, and kurtosis were calculated to check distribution of variables. 

As suggested by Anderson and Gerbing [47], in order to check the adequacy of the items to the identified dimensions, a Cronbach’s alpha was calculated and a confirmatory factor analysis (CFA) was run. In order to determine goodness of fit, factor loadings should be at least 0.4 [48], composite reliability (CR) should be above 0.70, and average variance extracted (AVE) above 0.50 [49].

A structural equation model (SEM) with latent variables was then calculated to examine the relationships between the variables described above and, in particular, to check Hypothesis 4. SEM is a second-generation statistical method that, in contrast to regression, allows for the simultaneous assessment of multiple independent and dependent constructs, including multistep paths and mediating effects. With SEM, the fit of the hypothesized model with data is generally evaluated by a series of indices; usually, an acceptable fit is indicated by relative χ^2^ (namely, χ^2^/df) below 5 [50], root-mean-square error of approximation (RMSEA) and its confidence interval less than 0.80 [51], standardized root-mean-square residuals (SRMR) lower than 0.08, and Comparative Fit Index (CFI), Normed Fit Index (NFI), and Tucker–Lewis index (TLI) greater than 0.95 [52]. 

We used partial least squares to assess model parameters. We used 10,000 bootstrapping samples to estimate standard errors [53]. CFA and SEM calculations were carried out using Amos [54]. 

### 3.4. Ethics

At inclusion, all participants received a web-based questionnaire and a covering letter (including a request for informed consent) providing information about the purpose and the voluntary character of participation in the study. Anonymous IDs were used for each participant, the completed questionnaires received by the researchers could never be linked to the identifying information, and anonymity was preserved at all times. The Ethical Review Board of the Department of Psychology at the Catholic University of Milan (Italy) approved the research protocol (protocol number: 12-2019).

## 4. Results

### 4.1. Socio-Demographics and Clinical Characteristics

The sample had an average age of 46.28 years (SD = 13.19) and were 67.1% females. Table 1 shows the distribution of the socio-demographical and clinical characteristics of the sample included in the study.

### 4.2. Descriptive Characteristics

The values of the mean, standard deviation, skewness, and kurtosis of every observed variable are showed in Table 2. No item shows excessive skewness or kurtosis (above 1 or below −1), hence normal distribution of data can be assumed. Correlation between study’s measures has also been computed and is shown in Table 3.

### 4.3. Measurement Model

Measurement model reliability was evaluated calculating Cronbach’s standardized α and performing a CFA including three latent variables using ML method. CR and AVE have been calculated for each construct and factor loadings have been calculated for each item. The measurement model is reported in Table 4.

### 4.4. Structural Model and Hypotheses Testing

Estimated fit indices seem to show a good model fit, in particular: χ^2^/df = 4.827 (*p* < 0.001); SRMR = 0.0326; RMSEA = 0.062 (LO90 = 0.055; HI90 = 0.068); CFI = 0.969; NFI = 0.962; TLI = 0.962.

Total effects between constructs have been calculated to check hypotheses 1, 2 and 3. As shown in Table 5, results support the existence of the hypothesized relations between constructs. In particular, autonomy-supportive healthcare climate has both a significant, positive effect on PE (β = 0.191; *p* < 0.001) and on health literacy (β = −0.141; *p* < 0.001); finally, PHE-S has a positive and significant effect on BHLS (β = −0.392; *p* < 0.001). Negative marks are due to the fact that BHLS scale has reverse scoring (higher scores mean lower literacy).

### 4.5. Mediating Effect and H4

Results described above show that the three assumptions required to demonstrate the mediating role of a variable between a predictor and an outcome are met: the predictor (HCCQ) has a significant effect on both the mediator (PHE-S) and the outcome (BHLS), while the mediator has a significant effect on the outcome as well.

**Figure 2:**
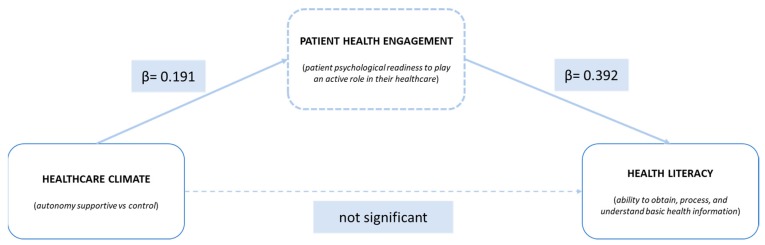
Results of structural equation modeling analysis: the full mediation effect of patient health engagement on healthcare climate in health literacy.

To check whether there actually is a mediating effect, direct and indirect effects of the predictor (HCCQ) on the outcome (BHLS) have been computed. Table 6 shows total, direct, and indirect effects of HCCQ on BHLS. Figure 2 shows the model with standardized betas.

Results show that when taking into consideration the indirect pathway, the role of HCCQ on BHLS is fully mediated by PHE-S, since the direct path that goes between HCCQ and BHLS becomes nonsignificant. Hence, we found the mediating effect of PHE-S on the HCCQ–BHLS relationship. Thus, H4 is confirmed by the data.

## 5. Discussion

Since health literacy plays a crucial role in chronic disease management, understanding the relationship between health literacy and the quality of healthcare climate may provide important insights for clinicians who care for such patient populations and may have important implications for the reduction of inequalities in the care of chronic conditions. To date, potential solutions to enhance patients’ health literacy skills have focused on improving the readability and understandability of medical documents or on adopting new technologies as a means to deliver information [55,56]. Although these efforts will surely lead to helpful changes in supporting patients in the acquisition of skills to obtain, process, and understand basic health information, our study considered other crucial variables and suggests that patients who are more engaged in their healthcare and are psychologically ready to be active players in the patient–doctor relationship are also more literate.

The major findings of this research are aligned with those of previous studies, highlighting the impacts of autonomy-supportive healthcare climate on patient’s health literacy and patient’s psychological readiness to engagement in the care process [32,33,57]. This study showed that autonomy-supportive healthcare climate has a positive correlation with the patient ability to obtain, understand, and use health information along their healthcare journey; These findings are consistent with the results of previous studies [7,58,59], proposing pathways of the effects of a patient–provider relationship oriented to support the patient’s autonomy on patient’s health literacy. This means that patients who perceive that their healthcare providers are likely to support their autonomy in managing their health have a better understanding of prescription labels, effectively interpret their health values or medication dosing schedules, and extract/criticize health information.

Among a diverse array of potential intervening variables in the relationship between healthcare climate and health literacy, this study found the mediating role of the Patient Health Engagement model (such as the measure of the patients’ psychological readiness to engage) in healthcare in this path. Our analysis demonstrated patient health engagement to be a critical construct. While an indirect pathway between autonomy-supportive climate and health literacy via Patient Health Engagement has been mentioned in the literature [32,60], empirical research has not reported on the subject. To our knowledge, the present study is the first to support the presence of an indirect relationship among those variables and to empirically demonstrate the role of patients’ psychological readiness to engage in this interaction. The study model suggests that the Patient Health Engagement model is crucial to the link between a healthcare climate oriented to support patient autonomy and health literacy. This means that the patient’s psychological readiness to play an active role during the healthcare encounters is a key factor in the influence of autonomy-supportive climate on the patients’ health literacy level.

In clinical practice, it is very important to stimulate and promote the patient’s ability to collect, understand and use health information. To achieve this, healthcare professionals should consider adopting relational strategies to support patients in adopting a partnership and autonomous role in the care process through the psychological acceptance of their health condition. For example, empowering the patient’s perceptions of his or her own ability to take control over his or her life regarding the disease and to find a “new normality” may be a useful strategy for improving patients’ health literacy [61]. For this reason, a healthcare model merely oriented to train healthcare professionals in improving communication skills to foster patient health literacy might be not enough to reach this goal. A medical education program aimed to support healthcare professionals in adopting new models of care oriented to interpret the medical encounter as a partnership setting where both clinicians and patient share all parts of the health decision-making process and play an active role in the care journey is warranted to effectively support individuals in becoming literate about their condition. Our study has a number of limitations. First, one of our main variables was patients’ reports of their physician’s relational and communication processes of care and not direct observations. This could be a source of bias related to social desirability effect. Second, we did not control the model for important confounders that we hypothesized could impact health literacy skills, such as age and educational level. For this reason, it is possible that our findings are a result of residual confounding. Furthermore, potentially confounding variables such as education were not considered in this study due to the fact that these variables were self-reported and very subject to bias. Further study should consider this limitation. Another limitation in this study is its use of a cross-sectional design, which means that, unlike a longitudinal design, causal relationships among study variables could not be determined. Finally, the measurement model of the items we used to assess health literacy was not completely adequate: the low alpha suggests that the measurement of health literacy may have been inaccurate. This could be due to an ineffective adaptation of the items to Italian language or culture. However, the overall goodness-of-fit of our model vouches for its validity. Further studies with more adequate measures should be carried out in order to corroborate the mediation relation found here.

## 6. Conclusions

The research points offer new insights into the science of health literacy and allow the healthcare provider to create new opportunities for promoting this crucial factor through the development of a healthcare climate aimed to sustain the patient’s effective psychological readiness to play an active role in his/her own healthcare. Particularly, the Patient Health Engagement model, by featuring four incremental psychological positions of engagement, motivation, and attitudes, appears valuable in generating insight about the way patients will react to different doctor–patient communication styles. Therefore, this study offered new insight about health literacy and autonomy-supportive interventions in the healthcare literature, providing key insights for future researches.

## Figures and Tables

**Table 1:** Sample characteristics.

Variables	*n*	%
Gender		
Female	676	67.1
Male	331	32.9
Age		
18–30	131	13.0
31–50	483	48.0
51–70	358	35.6
>70	35	3.4
Hospitalized last year		
Yes	188	18.7
No	819	81.3
Disease condition		
Cardiovascular disease	127	12.6
Thyroid disease	124	12.3
Autoimmune disease	117	11.6
Arthritis	100	9.9
Pulmonary disease	100	9.9
Cancer	98	9.8
Diabetes	75	7.4
Migraine	60	6.0
Multiple sclerosis	55	5.5
Gastrointestinal disease	20	2.0
Skin disease	17	1.7
Osteoporosis	13	1.3
Other diseases	101	10.0
Patient Health Engagement (PHE-S)		
Blackout	61	6.1
Alert	380	37.7
Adherence	458	45.5
Eudaimonic Project	108	10.7

**Table 2:** Mean, standard deviation, skewness, and kurtosis of the study’s measures.

Variables	Mean	Median	Std. Dev.	Skewness (S.E.)	Kurtosis (S.E.)
Autonomy-Supportive Healthcare Climate (HCCQ)					
Item 1	4.20	4	1.68	−0.33 (0.08)	−0.55 (0.15)
Item 2	4.88	5	1.63	−0.67 (0.08)	−0.15 (0.15)
Item 3	5.15	5	1.56	−0.80 (0.08)	0.19 (0.15)
Item 4	5.07	5	1.53	−0.77 (0.08)	0.21 (0.15)
Item 5	5.09	5	1.61	−0.81 (0.08)	0.09 (0.15)
Item 6	4.83	5	1.48	−0.54 (0.08)	−0.02 (0.15)
Patient Health Engagement (PHE-S)					
Item 1	4.28	5	1.48	−0.29 (0.08)	−0.31 (0.15)
Item 2	4.46	5	1.38	−0.27 (0.08)	−0.12 (0.15)
Item 3	4.54	5	1.55	0.14 (0.08)	−0.90 (0.15)
Item 4	4.44	5	1.63	−0.20 (0.08)	−0.53 (0.15)
Item 5	4.60	5	1.56	−0.07 (0.08)	−0.62 (0.15)
Health Literacy (BHLS)					
Item 1	1.30	1	0.99	0.24 (0.08)	−0.72 (0.15)
Item 2	1.35	1	1.06	0.55 (0.08)	−0.20 (0.15)
Item 3	1.40	1	1.13	0.36 (0.08)	−0.73 (0.15)

**Table 3:** Correlation between study’s measures.

Variables	Index	HCCQ	PHE-S	BHLS
HCCQ	Spearman’s rho	—		
	*p*-value	—		
PHE-S	Spearman’s rho	0.198	—	
	*p*-value	<0.001	—	
BHLS	Spearman’s rho	−0.136	−0.404	—
	*p*-value	<0.001	<0.001	—

**Table 4:** Measurement model.

Variables	Factor Loadings	Cronbach’s α	CR	AVE
Autonomy-Supportive Healthcare Climate (HCCQ)		0.927	0.84	0.48
Item 1	0.599			
Item 2	0.928			
Item 3	0.916			
Item 4	0.850			
Item 5	0.899			
Item 6	0.777			
Patient Health Engagement (PHE-S)		0.914	0.89	0.48
Item 1	0.798			
Item 2	0.839			
Item 3	0.822			
Item 4	0.828			
Item 5	0.840			
Health Literacy (BHLS)		0.636	0.64	0.39
Item 1	0.848			
Item 2	0.375			
Item 3	0.642			

CR: composite reliability; AVE: average variance extracted.

**Table 5:** Path estimates (β) for testing Hypotheses 1–3.

Hypothesis	Path	Std. Beta	*p*-Value
H1	HCCQ -> BHLS	−0.136	<0.001
H2	PHE-S -> BHLS	−0.393	<0.001
H3	HCCQ -> PHE-S	0.198	<0.001

**Table 6:** Total, direct, and indirect effects of HCCQ on BHLS.

Path	Std. Beta	*p*-Value
Total (HCCQ -> BHLS)	−0.136	*p* < 0.001
Direct (HCCQ-> BHLS)	−0.058	*p* = 0.152
Indirect (HCCQ -> PHE-S -> BHLS)	−0.078	*p* < 0.001
